# Selective Androgen Receptor Modulators in Women: What Do We Know, and What Is Still Missing

**DOI:** 10.3390/life16020359

**Published:** 2026-02-20

**Authors:** Veselin Vasilev, Katerina Georgieva, Maria Kraeva, Raina Ardasheva, Rumyana Etova, Nikolay Boyadjiev

**Affiliations:** 1 Department of Physiology, Medical Faculty, Medical University of Plovdiv, 4002 Plovdiv, Bulgaria; kng@plov.net (K.G.); boyad@plov.net (N.B.); 2 Department of Otorhinolaryngology, Medical Faculty, Medical University of Plovdiv, 4002 Plovdiv, Bulgaria; maria.kraeva@mu-plovdiv.bg; 3 Department of Medical Physics and Biophysics, Faculty of Pharmacy, Medical University of Plovdiv, 4002 Plovdiv, Bulgaria; rayna.ardasheva@mu-plovdiv.bg; 4 Department of Epidemiology and Disaster Medicine, Faculty of Public Health, Medical University of Plovdiv, 4002 Plovdiv, Bulgaria; rumyana.etova@mu-plovdiv.bg; 5 Research Institute, Medical University of Plovdiv, 4002 Plovdiv, Bulgaria

**Keywords:** SARMs, androgen receptor, women, androgens

## Abstract

Androgens and androgen receptor (AR) signaling influence many aspects of female physiology, including reproduction, musculoskeletal health, metabolism, and neurological regulation, yet are less studied than in males. Selective androgen receptor modulators (SARMs) were developed to provide tissue-selective anabolic effects with reduced androgenic side effects, but their effects in women are not well defined. This narrative review summarizes preclinical and clinical evidence on SARM use in female rodents and women, focusing on AR biology, tissue selectivity, therapeutic potential, and safety. A literature search of PubMed, Scopus, and Google Scholar identified relevant experimental and clinical studies addressing sex-specific AR signaling and SARM effects in females. Preclinical data indicate that SARMs can enhance sexual motivation and improve muscle and bone outcomes in ovariectomized models, with compound-dependent effects on reproductive tissues. Clinical studies in postmenopausal women demonstrate increases in lean body mass with generally limited androgenic effects, although functional benefits are inconsistent and alterations in lipid profiles and liver enzymes have been reported. Evidence also supports antitumor activity of AR-targeted SARMs in selected breast cancer subtypes. Overall, while SARMs show therapeutic potential in women, long-term safety and efficacy remain insufficiently characterized, warranting further sex-specific clinical investigation.

## 1. Introduction

Androgens, though traditionally considered male sex hormones, have essential and diverse functions in female physiology. In women, circulating androgens including testosterone, dehydroepiandrosterone (DHEA), and their precursors exert effects by binding to the androgen receptor (AR). Research has shown that ARs are widely expressed in many female tissues, where they influence key physiological processes such as reproductive and sexual function, cardiovascular health, bone and muscle maintenance, and neurological regulation [1]. ARs also play a role in the regulation of liver metabolism and adipose tissue function [1]. Despite their broad relevance, most studies on AR signaling have historically focused on male biology, leaving substantial gaps in understanding how modulation of these receptors affects women across different life stages [2].

Interest in selective androgen receptor modulators (SARMs) has renewed attention to AR biology in females. SARMs are synthetic, non-steroidal compounds engineered to preferentially activate the AR in muscle and bone while reducing the androgenic effects associated with testosterone or anabolic steroids [3]. Initially developed for conditions such as cachexia, osteoporosis, and muscle wasting, their effects in women remain largely unexplored. Most clinical trials have either focused on men or included mixed-sex populations, which limits the ability to draw conclusions specifically about women [4,5]. To date, female-specific research on SARMs is limited, beginning with a phase IIA randomized, placebo-controlled trial of MK-0773 in women over 65 years with sarcopenia, followed by several studies conducted in the context of breast cancer [6,7,8].

Beyond clinical research, AR modulation is attracting attention among women in fitness, aging populations, and those seeking alternatives to conventional hormone therapies. For example, SARMs such as ostarine have been identified in anti-doping tests in female combat athletes, even at low concentrations [9]. This trend emphasizes the need to understand both potential benefits and risks. Women may respond differently to SARMs due to variations in endogenous androgen levels, interactions between estrogen and androgens, and AR gene polymorphisms, which can alter pharmacokinetics, hormonal feedback, and side-effect profiles [10,11]. The potential adverse effects of androgen excess, including seborrhea, acne, hirsutism, menstrual irregularities, clitoral enlargement, voice deepening, infertility, and metabolic disturbances remain poorly characterized [12]. Understanding these unknowns is crucial for the safe and effective application of emerging therapeutics.

Given the increasing interest and limited female-focused evidence, a comprehensive review of the available data is warranted. This review aims to synthesize preclinical and clinical evidence on SARMs in female rodents and women, summarize reported adverse events, compare the AR in males and females, discuss the role of ARs in female physiology and identify critical knowledge gaps that must be addressed to develop safe, effective, AR-targeted therapies for female health.

## 2. Materials and Methods

This narrative review was conducted to synthesize and critically evaluate the current preclinical and clinical evidence on SARMs in female physiology and disease. A comprehensive literature search was performed using the electronic databases PubMed, Scopus, and Google Scholar. Searches were conducted using combinations of the following keywords: “selective androgen receptor modulators,” “SARMs women,” “androgen receptor sex differences,” “androgen receptor signaling,” “SARMs female rodents”. Additional references were identified by manual screening of bibliographies from relevant review articles and key primary studies.

The initial search yielded over 300 articles. Titles and abstracts were screened for relevance, followed by abstract and full-text review (where available) of selected publications. Studies were included if they met at least one of the following criteria: reported preclinical data on SARMs in female rodents (rats or mice), including ovariectomized (OVX) models; presented clinical data on SARM use in women, either as primary study populations or as sex-disaggregated analyses; examined sex-specific differences in AR, signaling, or function, with relevance to female physiology; addressed safety, pharmacology, or adverse effects of SARMs with potential implications for women.

No restrictions were applied regarding publication year, given the evolving nature of SARM development; however, priority was given to recent and high-quality studies. Only articles published in English were included. Data extraction focused on study design, population characteristics, SARM compound and dosing, duration of exposure, physiological or clinical outcomes, and reported adverse events.

As this is a narrative review, no formal risk-of-bias assessment or meta-analysis was performed. Instead, findings were synthesized qualitatively, with emphasis on identifying consistencies, discrepancies, translational limitations, and knowledge gaps in the existing literature, particularly with respect to female-specific responses and long-term safety considerations.

## 3. Results

### 3.1. Effects on Sexual Organs, Motivation and Libido

Several SARMs (S-22, S-23, S-26, S-27, S-28, S-30) administered for 14 days enhanced sexual motivation in ovariectomized rats, producing effects comparable to testosterone propionate [13]. These findings show the pivotal role of the AR in regulating female libido and suggest SARMs could be an alternative to steroidal testosterone for hypoactive sexual desire. In the same study, S-23, S-25 and S-26 treatments normalized ovariectomy-induced elevations in serum luteinizing hormone (LH) and follicle-stimulating hormone (FSH), while all tested SARMs (from S-22 to S-30) mitigated the ovariectomy-associated reduction in myometrial thickness (Table 1). Similarly, LGD-3303 increased sexual motivation toward male rats in OVX females, although this effect was observed only in animals that had been sexually active prior to the experiment. LGD-3303 also stimulated female behaviors that facilitate copulation [14] (Table 1).

Preclinical studies in OVX mice further demonstrate tissue-selective effects on the uterus and highlight the differing effects of individual SARM compounds. Ostarine (enobosarm, GTx-024) increased body weight, uterine weight, endometrial surface area, Ki67-positive stromal cells, and epithelial proliferation, whereas GTx-007 (Andarine) had minimal effects [15]. Ostarine’s uterotrophic effects closely mirrored those of dihydrotestosterone. Correspondingly, S-101479 administered for 12 weeks produced modest, dose-dependent increases in clitoral gland and uterine weights in OVX rats, but these were markedly less pronounced than DHEA and were not associated with overt histopathological changes, indicating reduced virilizing and uterotrophic activity consistent with tissue-selective AR modulation [16] (Table 1).

However, these findings are derived almost exclusively from short-term preclinical models, and controlled clinical studies evaluating sexual function, libido, and reproductive tissue safety in women are lacking. In particular, the long-term consequences of AR modulation on uterine and breast tissues remain insufficiently characterized.

### 3.2. Effects on Muscles and Body Composition

SARMs enhance muscle mass and function in both animal and human studies. In OVX rats, ostarine and ligandrol administered for 5 weeks increased muscle mass and capillary density [17]. Ostarine enhanced capillary density in gastrocnemius and longissimus muscles and increased citrate synthase (CS) activity, while ligandrol increased CS activity in gastrocnemius and lactate dehydrogenase (LDH) in longissimus, and at higher doses increased muscle weight and intramuscular fat. Both compounds showed moderate uterotrophic effects at high doses (4 mg/kg) [17] (Table 1). Other animal models using S-4 or LGD-2226 demonstrated similar results, increasing strength and preventing muscle loss in androgen-deficient rats and mice [18,19] (Table 1).

In humans, the SARM GSK-2881078 administered for 13 weeks increased lean body mass in healthy postmenopausal women and men with chronic obstructive pulmonary disease, though leg strength improvements were seen only in men [24] (Table 2). In earlier Phase I studies, GSK-2881078 given for up to 28 days (and up to 56 days in dose-escalation cohorts of 0.35–1.5 mg) produced dose- and duration-dependent increases in appendicular and total lean body mass in women, with greater responsiveness at lower doses compared with men, and increased thigh muscle volume by MRI, though physical performance outcomes were not formally assessed [5,25] (Table 2).

Ostarine (3 mg daily for 12 weeks) in healthy elderly men and postmenopausal women increased lean body mass by an average of 1.4 kg and improved functional performance, including stair-climbing capacity (+15–25%) [26] (Table 2). However, in patients with cancer-related muscle wasting, ostarine (1–3 mg for 113 days) increased lean body mass and stair-climbing capacity but did not improve hand grip strength [27] (Table 2). Similarly, MK-0773 (50 mg twice daily for 6 months) increased lean body mass in postmenopausal women with sarcopenia, although functional performance was not significantly enhanced [8] (Table 2).

Importantly, while SARMs consistently increase lean body mass, the clinical relevance of these changes remains uncertain, as functional improvements are inconsistent across studies. Data in frail populations, cancer patients receiving active therapy, and women with advanced disease are particularly limited.

### 3.3. Effects on Urinary Function and Stress Incontinence

In female OVX Sprague-Dawley rats, 4 weeks of GSK-2849466A treatment dose-dependently improved urethral continence. Low-dose treatment restored baseline urethral pressure, while high-dose treatment normalized both baseline pressure and sneeze-induced urethral response, preventing stress urinary incontinence. High-dose SARM also hypertrophied urethral striated and smooth muscle, without affecting bladder function, indicating a targeted effect on urethral tissues [20] (Table 1).

Other SARMs, including GTx-027 and ostarine, restored pelvic floor muscle mass in OVX mice models, suggesting potential utility in treating stress urinary incontinence [21] (Table 1). Clinically, low serum testosterone in postmenopausal women is associated with increased risk of stress urinary incontinence [31]. However, a phase 2 clinical trial of ostarine for stress urinary incontinence (NCT03241342) did not meet its primary endpoint, and two additional studies were terminated due to insufficient efficacy [28] (Table 2).

Although preclinical data suggests potential benefits of SARMs on pelvic floor musculature, clinical translation has been unsuccessful to date. The lack of efficacy in phase 2 trials highlights the need for improved patient selection, alternative dosing strategies, or reconsideration of this indication altogether.

### 3.4. Antitumor Effects

SARMs, including ostarine and GTx-027, exhibit antiproliferative activity in AR-positive breast cancer models (MDA-MB-231-AR, triple-negative) [32]. This effect likely involves inhibition of tumor progression pathways and suppression of paracrine factors such as IL-6 and MMP13 [32].

Clinically, the first response to a nonsteroidal SARM in ER+/PR+/AR+ metastatic breast cancer was reported in 2017 after multiple ER-targeted therapies failed [29]. A phase 2 study showed 24 weeks of enobosarm produced anti-tumor effects in ER-positive, HER2-negative advanced breast cancer [7] (Table 2). RAD140 (testolone, vosilasarm) also demonstrated clinical activity with favorable safety in AR+/ER+/HER2− metastatic or advanced breast cancer in an ongoing phase I/II study [30] (Table 2).

Nevertheless, clinical evidence remains preliminary, with small patient numbers, limited follow-up, and a lack of validated biomarkers to predict response. The interaction of SARMs with existing endocrine therapies and their long-term impact on breast cancer biology remain unresolved.

### 3.5. Bone Effects

SARMs improve bone mass, strength, and quality in androgen- or estrogen-deficient rodent models. In orchiectomized (ORX) male rats, 4 months of LGD2226 prevented bone loss, stimulated periosteal bone formation, improved cortical architecture, and enhanced levator ani muscle mass, with minimal effects on the prostate and seminal vesicles [19] (Table 1). S-40503 administered for 4 weeks in ORX males or 2 months in OVX females increased femoral bone mineral density (BMD) and biomechanical strength, demonstrating direct osteoanabolic activity with minimal impact on sex accessory tissues or surrounding muscle [22] (Table 1).

In aged OVX females, daily S-4 from day 90 to 210 restored whole-body and lumbar vertebrae BMD, improved cortical bone quality at the femoral midshaft, and increased skeletal load-bearing capacity [18] (Table 1). LGD-3303 showed anabolic effects on muscle and cortical bone in osteopenic females, with additive benefits when combined with alendronate [23] (Table 1). S-101479 increased femoral BMD and bone size in OVX rats [16] (Table 1). In the earlier mentioned MK-0773 study in postmenopausal women, a non-significant numerical improvement in bone mineral content was reported [8] (Table 2).

Despite robust preclinical osteoanabolic effects, clinical data supporting fracture risk reduction or long-term skeletal benefit in women are currently insufficient, limiting conclusions regarding the role of SARMs in bone health management.

### 3.6. Pharmacological Profile and Safety in Clinical Trials

In the abovementioned Phase II trial of ostarine, in both older men and postmenopausal women, the adverse events were mostly mild and occurred at rates comparable to placebo, with headache and back pain being the most frequent. Women did not show clinically relevant androgenic effects [26] (Table 2). Reversible elevations in liver enzymes occurred in some participants, and only one woman discontinued due to alanine aminotransferase (ALT) elevation [26] (Table 2). Sex hormone levels including testosterone, dihydrotestosterone, and estradiol remained largely stable in women, while small reductions in LH and FSH were seen at the higher dose (3 mg) [26] (Table 2). Sex hormone-binding globulin (SHBG) decreased at both 1 mg and 3 mg doses, consistent with the drug’s mechanism, and slight increases in hemoglobin and hematocrit were observed. high-density lipoprotein (HDL) cholesterol declined in a dose-dependent manner, while total cholesterol, LDL (low-density lipoprotein), triglycerides, and the total cholesterol/HDL ratio remained relatively unchanged [26] (Table 2).

Postmenopausal women treated with GSK-2881078 displayed a distinct hormonal and metabolic safety profile [25] (Table 2). HDL cholesterol decreased in a dose-dependent way, with women appearing particularly sensitive [25]. SHBG reductions were also notable, reflecting AR-mediated changes in hormone binding and availability [5,25] (Table 2). Other reproductive hormones including FSH, LH, estradiol, and progesterone remained largely unaffected, suggesting the drug’s endocrine effects were largely limited to SHBG and lipid parameters [5,24,25] (Table 2). Temporary increases in liver enzymes, mainly ALT, were observed but were reversible and not indicative of significant liver toxicity [5,24,25] (Table 2).

In the previously mentioned 6-month study of MK-0773 in older women with sarcopenia, the treatment was generally well tolerated. However, some participants experienced temporary increases in liver enzymes such as ALT and aspartate aminotransferase (AST). A few discontinued the drug, but these changes resolved after treatment was stopped. Modest increases in hemoglobin and hematocrit were observed, reflecting androgen-driven stimulation of red blood cell production [8] (Table 2). A slight rise in systolic blood pressure was noted, but no significant androgenic side effects such as acne or excess hair growth occurred. Overall, MK-0773 raised safety concerns primarily involving liver function and blood parameters rather than overt androgenic effects [8] (Table 2).

In the abovementioned phase I/II study with RAD140 in women with breast cancer, the SARM was generally well tolerated, with most treatment-emergent adverse events being mild to moderate [30] (Table 2). The most common adverse effects were transient elevations in liver enzymes, nausea, and anemia, with grade ≥3 ALT increases occurring in about 20% of patients but no dose-limiting toxicities or treatment-related deaths [30] (Table 2). Although ostarine has been shown to slightly increase hemoglobin and hematocrit in healthy men and women [26], anemia was observed in women with advanced breast cancer treated with RAD140. This difference likely reflects the underlying disease state and its complications, including anemia of chronic disease, bone marrow suppression, or prior treatments which can mask or outweigh the erythropoietic effects of SARMs. Liver enzyme elevations were typically asymptomatic, occurred early in treatment, and were manageable with dose interruption or reduction (Table 2).

## 4. Discussion

### 4.1. Sex Differences in AR Signaling

The AR is a nuclear receptor that acts as a transcription factor. Upon binding to androgens such as testosterone or dihydrotestosterone, AR triggers the expression of target genes. The AR gene is located on the X chromosome [33]. While the AR protein is structurally identical in both males and females, its expression, activation, and tissue-specific sensitivity show significant sex differences. These differences are critical for understanding AR’s physiological roles and the effects of SARMs.

One key sex difference lies in circulating sex steroid levels: males typically have much higher testosterone concentrations than females [34]. Since AR activation depends on androgen binding, lower endogenous androgen levels in females generally lead to less pronounced AR-mediated signaling under normal conditions [34]. However, sex differences in AR signaling are not solely hormone dependent. In vitro studies of human skeletal muscle cells reveal that male and female cells respond differently to identical testosterone levels, exhibiting distinct patterns of gene regulation related to muscle contraction and sarcomere formation [35]. This demonstrates intrinsic, cell-level differences in AR signaling. Similarly, tissue-specific differences are observed in the brain, where men show stronger AR immunoreactivity than women in regions such as the mammillary bodies of the hypothalamus [36].

Males generally have higher AR protein levels in many tissues, including macrophages [37]. AR activity is particularly high in male-specific tissues such as the testes, prostate, and seminal vesicles, whereas in females, strong AR activity is found in the ovaries, uterus, omentum, and mammary glands [38]. AR regulation also differs between sexes. In skeletal muscle, women show higher phosphorylation of AR at specific sites despite lower overall receptor levels [39]. In males, AR content is closely associated with muscle size, whereas in females, the relationship between AR levels and muscle mass is different [40].

Functionally, AR plays a central role in reproductive organs, sexual behavior, muscle mass, and bone health in males, whereas in females it contributes more to bone metabolism, muscle function, and other non-reproductive roles [41]. Under normal conditions, AR-mediated androgen signaling contributes less to energy homeostasis, adipose tissue regulation, and metabolic control in women than in men [42]. Nevertheless, in pathological or pharmacological contexts such as elevated androgen exposure, hormone therapy, or exogenous androgen use AR activation in women can alter adiposity, fat distribution, and insulin sensitivity, potentially leading to adverse metabolic outcomes [42]. These sex-specific differences are particularly relevant when considering the effects of SARMs, as their metabolic and health impacts may differ substantially between men and women.

### 4.2. AR Function in Female Physiology

In women, androgens are primarily produced by the ovaries and adrenal glands, with levels fluctuating throughout the reproductive lifespan, showing cyclical variations during the menstrual cycle and a gradual decline with aging [1]. AR is expressed in several reproductive tissues, including ovaries, uterus, fallopian tubes, and broader reproductive tract tissues, suggesting a role in maintaining reproductive homeostasis [43]. Within the ovary, AR is present in theca cells, granulosa cells, and oocytes, implicating androgen signaling in follicular development, steroidogenesis, and fertility [44]. These localizations are functionally significant, as genetic models show that AR deficiency disrupts follicle maturation and estrous cyclicity, highlighting its critical role in ovarian function and female fertility [45].

Beyond steroid production, AR regulates genes involved in gonadotropin responsiveness and FSH signaling, both crucial for ovulation [45]. AR expression is dynamically regulated across the menstrual cycle in the endometrium, contributing to stromal cell differentiation essential for implantation and early pregnancy. Disruptions in AR-mediated androgen signaling, as observed in polycystic ovary syndrome, can impair reproductive outcomes [46]. Aberrant AR signaling has also been implicated in gynecological disorders; for example, AR expression in breast cancer intersects with estrogen receptor pathways, potentially influencing tumor progression and therapy resistance [47]. Despite its recognized importance, the precise mechanisms by which AR regulates female physiology, particularly in concert with estrogen and other hormones, remain under investigation [48].

Further than reproduction, AR contributes to multiple systemic processes. In skeletal muscle and bone, it helps maintain muscle mass and bone density, supporting overall musculoskeletal health [49]. Its presence in adipose tissue and the liver suggests roles in metabolic regulation, lipid handling, and insulin sensitivity, areas of growing interest in metabolic health [50]. AR expression in various brain regions further points to involvement in cognitive, behavioral, and neuroendocrine functions, though these roles are less well-characterized [51].

### 4.3. Tissue Selectivity of SARMs

Understanding these sex-specific roles of AR provides context for the development of SARMs. The selectivity of SARMs is achieved through multiple mechanisms. In anabolic tissues, SARMs act as full or partial agonists of the AR, whereas in androgenic tissues they function as partial agonists or antagonists, reducing adverse effects. A critical factor underlying this tissue specificity is the differential recruitment of coregulator proteins to the AR; SARMs induce distinct receptor conformations that influence whether coactivators or corepressors bind, and the distribution of these coregulators varies across tissues [52].

Furthermore, most nonsteroidal SARMs are not metabolized by 5α-reductase into dihydrotestosterone, preventing localized receptor hyperactivation associated with prostate complications seen with testosterone and anabolic steroids. The inability of most SARMs to undergo aromatization is particularly important for women, as it reduces estrogen-related side effects and hormonal fluctuations [53].

### 4.4. Safety Concerns in SARM Use

Despite these mechanistic advantages, SARMs remain investigational compounds, and none are approved by the U.S. Food and Drug Administration for human use. Regulatory agencies, including the FDA and USADA, have issued warnings regarding potential health risks associated with their use [54]. Evidence on SARM safety, particularly in women, remains limited. While preclinical studies have reported clitoral enlargement and uterotrophic effects (i.e., uterus growth), corresponding clinical data in women are scarce. A 2023 review identified 20 published reports of adverse events associated with SARM use in humans, including drug-induced liver injury manifesting as cholestatic and hepatocellular damage, as well as jaundice [55]. Notably, all reported cases involved male patients, underscoring the lack of sex-specific safety data.

Emerging concerns also relate to potential cardiovascular risks. AR overstimulation, particularly in the context of misuse or abuse, may negatively affect the renin–angiotensin system, lipid metabolism, vascular smooth muscle function, inflammatory pathways, and platelet activity, collectively increasing cardiovascular disease risk [56]. Supporting this concern, a case of acute myocarditis following SARM intake has been documented, again in a male patient [57]. Additionally, a case report in an otherwise asymptomatic weightlifter revealed altered liver function, disruptions in lipid metabolism, and hormonal imbalance following SARM exposure [58]. Although these reports predominantly involve men, they highlight important safety signals that warrant careful consideration, particularly given the absence of female-specific clinical data.

### 4.5. Clinical Implications and Remaining Gaps

The desired profile of SARMs in women, as originally conceptualized in 1999, emphasizes the provision of therapeutic anabolic benefits without androgenic side effects [59]. Ideally, SARMs would support or enhance sexual function, promote bone density, and maintain or increase lean muscle mass, thereby helping preserve strength and skeletal health, particularly in postmenopausal women. At the same time, these agents should avoid virilization, cause minimal fluid retention, and remain neutral with respect to cardiovascular risk factors, preserving the protective effects of estrogen. A critical requirement is hepatic safety, with no clinically significant elevations in liver enzymes, to permit long-term clinical use [59].

Importantly, most of the mechanistic and efficacy data summarized above derive from short-term preclinical models, and their direct extrapolation to human physiology particularly in women should be approached with caution. Species differences in androgen metabolism, endocrine feedback loops, and tissue-specific AR signaling may substantially modify the effects observed in rodent models.

Although some studies report gains in lean body mass, these do not always translate into meaningful functional improvements in real-world measures, such as walking speed or stair-climb power. Key intended advantages, including robust bone protection and complete avoidance of virilization, are still not fully proven. Most human trials are short-term, typically lasting only a few weeks to months, with only one study extending to six months. Consequently, long-term safety data including effects on hormonal feedback, fertility, menstrual and reproductive health, and metabolic and cardiovascular outcomes remain largely unknown. Most critically, unresolved cardiometabolic and liver safety concerns continue to pose significant barriers to broader clinical use and regulatory approval. Nevertheless, the antitumor properties of SARMs remain under investigation and may represent a potential future therapeutic avenue.

Preclinical studies, especially in ovariectomized rodent models, only partially reflect human female physiology. Differences such as the gradual hormonal changes of natural menopause, estrogen interactions, and complex endocrine feedback mechanisms make translation of animal findings to women uncertain. Furthermore, there is limited understanding of how individual factors such as age, endogenous hormone status (pre- versus post-menopausal), genetic polymorphisms (e.g., AR variants), estrogen-androgen interactions, and lifestyle factors like diet and exercise affect responses to SARMs in women. These gaps highlight the need for larger, longer-term, and more inclusive studies to fully evaluate the efficacy, safety, and real-world benefits of SARMs in female populations.

## 5. Conclusions

SARMs represent a promising but still incompletely defined class of compounds for use in women. Current evidence indicates that SARMs can exert tissue-selective androgen receptor activity with potential clinical relevance in several contexts. However, these conclusions are largely based on preclinical data short-term clinical studies and should be interpreted cautiously. Early clinical data suggest that certain SARMs may achieve antitumor activity in selected breast cancer populations while avoiding many of the virilizing effects associated with conventional androgens.

Beyond oncologic applications, SARMs have demonstrated consistent anabolic effects on lean body mass and bone parameters in preclinical models and short-term clinical studies in postmenopausal women and older adults. However, gains in body composition do not uniformly translate into functional improvements, underscoring an important gap between surrogate endpoints and clinically meaningful outcomes. Evidence supporting benefits in other female-specific conditions, such as sexual dysfunction or stress urinary incontinence, remains limited and, in some cases, inconclusive.

Despite encouraging signals, substantial gaps remain. Long-term safety data in women are lacking, particularly with respect to hepatic, cardiometabolic, and reproductive outcomes. Most human studies are of short duration, include small sample sizes, and are not designed to address sex-specific endpoints. In oncology, unanswered questions remain regarding optimal patient selection, predictive biomarkers of response, interactions with established endocrine therapies, and the long-term consequences of AR modulation in breast tissue.

In summary, while SARMs offer an attractive theoretical profile for women, the current evidence base remains insufficient to support broad clinical use. Addressing the critical gaps in long-term safety, functional efficacy, and disease-specific outcomes through rigorously designed, sex-specific clinical trials will be essential to define the true therapeutic potential of SARMs in female populations.

## Figures and Tables

**Table 1:** Preclinical effects of selective androgen receptor modulators (SARMs) in female and mixed-sex animal models.

SARMs	Model	Main Findings	Reference Numbers
Various SARMs (S-22 to S-30)	OVX rats	↑ Sexual motivation; normalized LH/FSH; ↑ myometrial thickness	[13]
LGD-3303	OVX rats	↑ Sexual motivation and proceptive behaviors (experience-dependent)	[14]
Ostarine	OVX mice	Uterotrophic effects similar to dihydrotestosterone	[15]
Andarine	OVX mice	Minimal uterine effects	[15]
S-101479	OVX rats	Modest uterine/clitoral effects; reduced virilization	[16]
Ostarine, Ligandrol	OVX rats	↑ Muscle mass, capillary density; mild uterotrophy at high dose	[17]
LGD-2226, S-4	ORX/OVX rodents	Prevented bone loss;↑bone strength	[18,19]
GSK-2849466A	OVX rats	Improved urethral continence without bladder effects	[20]
Ostarine, GTx-027	OVX mice	restored pelvic floor muscle mass	[21]
S-40503	ORX/OVX rats	increased femoral bone mineral density and biomechanical strength,	[22]
LGD-3303	OVX rats	anabolic effects on muscle and cortical bone	[23]

Abbreviations: OVX—ovariectomized; ORX—orchiectomized; LH—luteinizing hormone; FSH—follicle-stimulating hormone; ↑—increased.

**Table 2:** Clinical effects and safety of selective androgen receptor modulators (SARMs) in women and mixed-sex human studies.

SARM	Population	Main Efficacy Outcomes	Safety Observations	Reference Numbers
GSK2881078	Healthy postmenopausal women and men (NCT02567773) Postmenopausal women and men with COPD (NCT03359473) Healthy older men and women (NCT not available)	Increased lean body mass	Decreased HDL and SHBG; reversible ALT elevation	[5,24,25]
Ostarine	Healthy elderly men and postmenopausal women (NCT not available), as well as age-matched individuals with cancer (NCT00467844)	Increased lean mass and improvement of physical performance	Mild adverse events; dose-dependent HDL reduction	[26,27]
MK-0773	Postmenopausal women with sarcopenia (NCT00529659)	Increased lean mass without functional improvement	Transient ALT/AST elevation; increased hemoglobin	[8]
Ostarine	Postmenopausal women with stress urinary incontinence (NCT03241342)	No significant clinical efficacy	Trial termination for insufficient efficacy	[28]
Ostarine	A 65-year-old woman with ER+/PR+, HER2− breast cancer (Case report)	clinical benefit for 11 months before meeting criteria for progression	Facial acne, fatigue	[29]
Ostarine	Postmenopausal women with ER+/HER2− breast cancer (NCT02463032)	Anti-tumor activity	Generally well tolerated: elevations of ALT, AST,	[7]
RAD140	Postmenopausal women with AR+/ER+/HER2− breast cancer (NCT05573126)	Clinical activity observed	Manageable liver enzyme elevations	[30]

Abbreviations: ALT—alanine aminotransferase; AST—aspartate aminotransferase; HDL—high-density lipoprotein; SHBG—sex hormone-binding globulin; COPD—Chronic obstructive pulmonary disease.

## Data Availability

No new data were created or analyzed in this study. Data sharing is not applicable to this article.

## Abbreviations

The following abbreviations are used in this manuscript:

SARMs	Selective Androgen Receptor Modulators
AR	Androgen Receptor
DHEA	Dehydroepiandrosterone
SHBG	Sex Hormone–Binding Globulin
LH	Luteinizing Hormone
FSH	Follicle-Stimulating Hormone
OVX	Ovariectomy
ORX	Orchiectomy
ALT	Alanine Aminotransferase
AST	Aspartate Aminotransferase
LDL	Low-Density Lipoprotein
HDL	High-Density Lipoprotein
COPD	Chronic obstructive pulmonary disease
